# Numerical investigation on the aerodynamic efficiency of bio-inspired corrugated and cambered airfoils in ground effect

**DOI:** 10.1038/s41598-022-23590-2

**Published:** 2022-11-09

**Authors:** G. R. Abdizadeh, M. Farokhinejad, S. Ghasemloo

**Affiliations:** 1 Aerospace Engineering Department, Amirkabir University of Technology, Tehran, Iran; 2 Department of Mechanical and Aerospace Engineering, Tarbiat Modares University, Tehran, Iran; 3 Faculty of Aerospace Engineering, Malek Ashtar University of Technology, Tehran, Iran

**Keywords:** Fluid dynamics, Engineering, Aerospace engineering

## Abstract

This research numerically investigates the flapping motion effect on the flow around two subsonic airfoils near a ground wall. Thus far, the aerodynamic efficiency of the dragonfly-inspired flapping airfoil has not been challenged by an asymmetric cambered airfoil considering the ground effect phenomenon, especially in the MAV flight range. The analysis is carried out on the basis of an unsteady Reynolds-averaged Navier-stokes (URANS) simulation, whereby the Transition SST turbulence model simulates the flow characteristics. Dragonfly-inspired and NACA4412 airfoils are selected in this research to assess the geometry effect on aerodynamic efficiency. Moreover, the impacts of Reynolds number (*Re*), Strouhal number (*St*), and average ground clearance of the flapping airfoil are investigated. The results indicate a direct relationship between the airfoil’s aerodynamic performance ($C_l$/$C_d$) and the ground effect. The $C_l$/$C_d$ increases by reducing the airfoil and ground distance, especially at $h_{0}=c$. At $Re=5\times 10^4$, by increasing the St from 0.2 to 0.6, the values of $C_l$/$C_d$ decrease from 10.34 to 2.1 and 3.22 to 1.8 for NACA4412 and dragonfly airfoils, respectively. As a result, the $C_l$/$C_d$ of the NACA4412 airfoil is better than that of the dragonfly airfoil, especially at low oscillation frequency. The efficiency difference between the two airfoils at *St*=0.6 is approximately 14%, indicating that the $C_l$/$C_d$ difference decreases substantially with increasing frequency. For $Re=5\times 10^3$, the results show the dragonfly airfoil to have better $C_l$/$C_d$ in all frequencies than the NACA4412 airfoil.

## Introduction

Bio-inspired innovation in engineering design has attracted significant attention in recent years, especially in Micro-Air Vehicles (MAV), for improving flight performance^1,2^. Nature gives us outstanding examples of flight from various points of view, such as optimal energy consumption at high dexterity motions, low noise, stability, and well mobility. Insect flight is one of the most sophisticated flying types^3–5^. Dragonflies, one of the many insects with exceptional flying ability, have distinct flight characteristics and great maneuvering abilities^6^. One of the advantages of dragonfly flight is that it flies close to the surface of the water to lay eggs or hunt other insects^7^. They may flap their wings up and down, as well as rotate them forth and back on an axis. Dragonflies may move straight up or down, fly backward, stop, and hover^6^. Their wings possess a corrugated cross-section with a camber. This corrugated pattern is critical in the ultra-lightweight construction of the wing structure, with great aerodynamic performance. The wings’ corrugation structure has been widely researched. The configuration changes when the spanwise and chordwise directions are changed. Aside from the weight issue, it enhances flight performance in various ways, including stress absorption against spanwise bending, permitting torsion, and creating a camber^8–10^. Aerodynamic force experiments reveal that, compared to the smooth-surfaced airfoil and flat plate, the corrugated airfoil could generate greater lift and delay airfoil stall to a significantly higher angle attack for low Reynolds number (*Re*) flight applications less than $10^5$^10^. The ability of the dragonflies, one of the fastest insects, to glide and perform adroit maneuvers during the flight caught the eye of engineers and scientists to improve the MAVs performance. One of the interesting phenomena of flight that has attracted remarkable attention is the ground effect on the aerodynamics of flapping wings. It has been demonstrated that the flying in the ground effect provides notable advantages in thrust and efficiency^11^.

One of the biggest advantages of the MAVs is their ability to carry out missions in a confined space, such as flying for reconnaissance, search-and-rescue, inspection, and monitoring in the oil and gas industry. However, in environments that are so crowded and full of obstacles, these MAVs will inevitably fly near a substrate, experiencing a wall effect. A similar scenario in the daily flight of small birds and tiny insects, which use flapping wings as their source of lift, is the flight in bushes full of leaves and branches^12^. These situations will also project a ground effect on the aerodynamics of flapping wings. Determining the ground impact on the aerodynamics of flapping wings is necessary for understanding the behaviors of near-ground flight in insects and developing flapping MAVs^13^.

The ground impact is the enhanced lift and reduced drag produced by an airfoil or other flying object when the lift-generated surface is one wingspan or less above the ground or water’s surface^14,15^. The blockage of a wing-tip vortex expansion caused by the ground is one of two mechanisms accountable for this particular phenomenon. A wing-tip vortex is also known as a lift-induced vortex since it alters the airflow around a wing and reduces the efficiency of lift production; this blockage considerably reduces drag and raises lift,similar to increasing the efficient angles of attack^16,17^. Flying nearby the ground, in addition to blocking vortex expansion, raises pressure on the lower wing surface and the ground since air is packed between the airfoil and the ground; this is known as the ram or cushion impact^16^. The research has primarily focused on fixed wings, and few investigations have been published on flapping wings associated with ground impact. Ahmed et al.^18^ comprehensively reviewed the aerodynamic properties of the NACA4412 airfoil at various Angles of Attack (AoA) from 0 to 10 deg and ground clearance of the trailing edge from 5% of the chord to 100% at a $Re=3\times 10^5$. Their results indicated that the drag coefficient ($C_d$) was higher near the ground for all AoAs, mainly owing to the lower surface pressure distribution. The findings demonstrated that at the minimum ground clearance, a strong suction impact on the lower surface at an angle of attack of 0 deg made laminar separation considerably ahead of the trailing edge. Importantly, for all angles of attack, a reduction of upper surface suction was detected as the airfoil reached the ground. The lift reduced with decreasing ground clearance for angles up to 4 deg, while, for higher angles, it raised owing to higher pressure on the lower surface. The drag was higher near the ground for all angles examined, mostly because of the lower surface pressure distribution modification. Qu et al.^19^ designed a NACA4412 airfoil landing process with a pitch angle of 4$^{\circ }$ and a flight path angle of 4$^{\circ }$ utilizing a finite volume technique. They revealed that the lift in dynamic ground effect (DGE) was greater than static ground effect (SGE); they also explained the phenomenon of lift amplification in DGE.

Using an Immersed Boundary-Lattice Boltzmann Method (IB-LBM), Gao and Lu^20^ explored the ground effect during the regular hovering flight of elliptic foil. The ground effect was found to alter thrust under three regimes: force increase, decrease, and recovery. This study offered physical insight into an understanding of aerodynamics and flow structures for insect typical hovering flight with a ground effect, as well as flying mechanics related to insect perching on the body. Su et al.^21^ studied the flapping-flying model relying on the actual behaviors of the birds under the ground effect. They determined that flapping close to the ground increases lift force by 47% while decreasing drag force by 20%. Wu et al.^22^ evaluated the contribution of the ground effect on the flapping insect wing in forward flight. In order to simulate the motion of the insect wing cross-section, a standard NACA0012 airfoil with harmonic plunge and pitch rotation was used. The IB-LBM was employed for numerical simulation at Re =150. According to the findings, the ground influenced both force behaviors and flow patterns. Whenever the foil was put near the ground, there was a significant decrease in drag and an increase in lift compared with the scenario with no ground. Furthermore, the vortex formed by the foil interacted with that caused by the ground. As the frequency of oscillation rose, the vortex interaction became increasingly more. Accordingly, the vortices might be compressed to oblate shape and propagate obliquely in the wake. Wu et al.^23,24^ numerically simulated the power extraction mode of the NACA0015 airfoil close to the ground. The simulation was performed at a Re of 1100, with a two-dimensional laminar flow system and a harmonic plunge and pitch rotating motion imposed. The IB-LBM was used to perform numerical simulations. The findings indicated that the ground influenced both force behavior and power extraction performance. In comparison to the case with no ground effect, the airfoil situated close to the ground enhanced power extraction efficiency. Because of the ground effect, the maximum efficiency was improved by 28.6%. Mivehchi et al.^25^ experimentally studied the ground effect for propulsive flapping NACA0012 airfoil operating vicinity ground at $Re= 2.1\times 10^4$. The results demonstrated that the mean distance from the wall significantly impacted the measured mean lift and thrust on the airfoil.

Lin et al.^26^ evaluated an asymmetrical heaving motion impact on the aerodynamic efficacy of a flapping NACA0012 airfoil close to a wall using 2D numerical simulations. The results indicated that the mean thrust monotonically rose as the foil gradually got close to the wall. Simultaneously, the mean lift first increased and then reduced suddenly. When the foil was very close to the wall, the mean lift even turned negative. Reducing the time of the upstroke increased the mean thrust coefficient. Younsi et al.^27^ examined the impact of the ground on an elliptic airfoil experiencing two hovering modes, “water treading” and “normal.” The unstable, laminar, incompressible Navier-Stokes equations were solved using the finite volume approach at Re=100. The findings confirmed that the rapid pitching up and delayed stall processes linked with the leading-edge vortex mechanisms accounted for the majority of lift production in both hovering modes. In the “normal” mode, however, two peaks in the lift coefficient were found at each stroke; this indicates the presence of the wake-capture system. The impact of ground clearance on the two hovering modes was determined to vary in terms of energy consumption and lift force variations. Zhu et al.^28^ studied the ground effect on the energy extraction qualities of a flapping NACA0015 airfoil at Re= 0.5-500. For variations in the oscillation frequency, the impacts of Re, average ground clearance, and effective angle of attack on the power extraction efficiency of a flapping airfoil were explored. The ground effect could substantially improve a flapping airfoil’s power extraction efficiency, and the ideal average ground clearance could be established to assure the best extraction. Sarbandi and Naderi^29^ numerically investigated the flapping motion of bio airfoil and a NACA0015 airfoil considering ground effect at Re=1100. The mean distance from the center of rotation of the airfoil to the ground surface ranged from 1.25 to 4 times the chord length of the airfoil; also, the motion’s lower frequency was set to 0.1 or 0.2. They determined that the power extraction efficacy of the Bio airfoil reduced when the mean distance to the ground increased, whereas it just marginally changed for the NACA0015 airfoil. The Bio airfoil outperformed the NACA0015 airfoil in the power extraction regime. Only on Bio airfoils does the leading edge vortex increase power extraction efficiency due to ground assistance. The propulsion effectiveness of both airfoils diminished as the distance to the ground increased, with the NACA0015 airfoil having a higher efficacy than the Bio airfoil. Tumse et al.^30^ investigated the aerodynamic efficiency and vortical flow structure of a delta wing during take-off and landing stages. Because of the incomplete development of vortices, the ground reduced the size of the peak values of main and secondary vortices. The ground effect induced the leading-edge vortex to travel outboard in a spanwise direction, increasing the size of the vortices. Moreover, the ground caused a drop in Strouhal number (*St*) because vortex generation was slowed. The delta wing’s lift and drag coefficients increased as it descended from the unbounded flight region towards the ground effect region. Eventually, it was determined that it rose by reducing the distance between the ground and the wing and that the rise was significantly more effective at lower angles of attack.  Table 1 presents research works about the oscillating airfoils near the ground. To clarify the roadmap for the present study, the motion type, the fluid flow regime, the applied solution method, and the geometry used in these researches were summarized in  Table 1.

**Table 1:** A brief look at the noteworthy researches about the influence of ground effect on aerodynamics.

Authors	Adopted mode	Motion	Method	Re	h/c	St
Ref.^18^	NACA4412	Static	Experimental	$3\times 10^5$	0.05-1	–
Ref.^19^	NACA4412	Static	Numerical (FVM)	$3\times 10^5$	0.05-1	–
Ref.^20^	Elliptic	Flapping	Numerical (IB-BLM)	100	1-5	0.25
Ref.^21^	Bird	Flapping	Numerical (FVM)	$2\times 10^4$	1.026,1.46, 1.9	0.1
Ref.^22^	NACA0012	Flapping	Numerical (IB-BLM)	150	1-5	0.1-0.5
Ref.^23,24^	NACA0015	Flapping	Numerical (IB-BLM)	1100	1-5	0.05-0.25
Ref.^25^	NACA0012	Flapping	Experimental	$2.1\times 10^4$	1.33-6	0.3-0.5
Ref.^26^	NACA0012	Flapping	Numerical (FVM)	500	1.2-2.5	0.25-0.75
Ref.^27^	Elliptic	Flapping	Numerical (FVM)	100	1-5	0.18
Ref.^28^	NACA0015	Flapping	Numerical (FVM)	500, 1100, $1.1\times 10^4$, $5\times 10^5$	1.8-34	0.02-0.1
Ref.^29^	NACA0015& Dragonfly	Flapping	Numerical (FVM)	1100	1.2-4	0.06
Ref.^30^	Delta wing	Static	Experimental	$1.573\times 10^5$	0.1-0.7	–

As seen above, most studies on flapping motion considering the ground effect phenomenon have been on symmetric airfoils such as NACA0012, NACA0015, and elliptic airfoils. Although all these airfoils are known in the industry, bio-inspired ones (e.g., the dragonfly airfoil) have been shown to have excellent aerodynamic performance compared to those of conventional in the vicinity of the ground. Therefore, one of the key factors in flapping airfoils and the ground effect process is the airfoil shape; As a result, choosing a proper airfoil is a crucial task in the design stage.

In addition, it has already been proven that the well-known cambered NACA4412 airfoil prevents the negative ground effect due to the nearly flat bottom surface that happens with extreme camber or when venturi flow is formed beneath the airfoil^31^. Therefore, this airfoil close to the ground has good aerodynamic performance due to its geometric shape. However, in previous studies, the flapping motion of the NACA4412 airfoil near the ground has not been investigated.

As mentioned, due to the geometrical shape of the dragonfly wing and the NACA4412 airfoil, their aerodynamic performance near the ground is well. However, their geometry has been emphasized in relatively few studies. To the best of our knowledge, the aerodynamic performance of dragonfly and NACA4412 airfoils with flapping motion considering the ground effect, has never been compared before in any scientific work. Further, the dragonflies and MAVs usually fly at a Re ranging from $10^3$ to $10^5$. However, most previous studies have focused on small *Re* and laminar flow.

In a nutshell, the present study compared the impact of the ground effect on the flapping dragonfly and NACA4412 airfoils. The simulation was done at Re of $5\times 10^3$ and $5\times 10^4$, using the 2D incompressible Unsteady Reynolds-Averaged Navier-Stokes (URANS) technique. The turbulence model was implemented using the Transition SST model. The Reynolds number and average ground clearance ($c\le h_{0}\le 5c$) impacts on the aerodynamic coefficient of the flapping airfoils were examined in relation to variations in the oscillation frequency ($0.2\le St\le 0.6$).

## Numerical model

### Governing equation and turbulence model

The mean flow over airfoil is assumed to be incompressible, two-dimensional and turbulent and governed by URANS equations given below,

1 $$\begin{aligned}{} & {} \frac{\partial u_i}{\partial x_i}=0 \end{aligned}$$

2 $$\begin{aligned}{} & {} \frac{\partial u_i}{\partial t} + u_j \frac{\partial u_i}{\partial x_j} = -\frac{1}{\rho } \frac{\partial P}{\partial x_i} + \upsilon \frac{\partial ^{2} u_i}{\partial x_i \partial x_j} - \frac{\partial \overline{u_{i}u_j}}{\partial x_i} \end{aligned}$$

Typical turbulence models in this approach are the $k-\epsilon$ or $k-\omega$ models in their different forms. Flow fields predicted by $k-\epsilon$ models are generally inaccurate under adverse pressure gradients and boundary layer separations^32^, and these models are therefore not recommended for application to pitching airfoils where both effects are of critical importance. Although this downside can be remedied by employing an equation for the specific dissipation rate $\omega$ instead of the turbulent dissipation $\epsilon$, the drawback of the standard $k-\omega$ model is that the solution is too sensitive to free stream values of *k* and $\omega$ outside the shear layer^33,34^. The $k\omega -SST$ model is designed to avoid such free stream oversensitivity. The Transition SST model (also known as the $\gamma$-$Re_{\theta t}$ model) is based on the combination of the $k\omega -SST$ model with two other correlation-based transport equations, one for the intermittency $\gamma$ and one for the transition momentum thickness Reynolds number $Re_{\theta t}$^32^. For wall-bounded flows, this model is better able to predict the laminar-turbulent transition. Hence, in this study, the Transition SST model is used for the 2D URANS calculations.

This turbulence model is obtained by adding two additional transport equations to $k\omega -SST$, one for intermittency $\gamma$ and the other for the transition Reynolds number $\widetilde{Re_{\theta t}}$. The transport equation for the intermittency $\gamma$ is well-defined as^35^:

3 $$\begin{aligned} \frac{\partial \rho \gamma }{\partial t} + \frac{\partial (\rho U_j \gamma )}{\partial x_j} = P_{\gamma 1} - E_{\gamma 1} + P_{\gamma 2} - E_{\gamma 2}+ \frac{\partial }{\partial x_j}\left[ \big (\mu +\frac{\mu _t}{\sigma _t}\big )\frac{\partial \gamma }{\partial x_j} \right] \end{aligned}$$

The model holds that rapid growth means flow separation. With the increase in turbulent viscosity ratio, the growth of tends to be flat^35,36^. The intermittent factor $\gamma _{sep}$ specially designed for the transition of separated flow is expressed as:

4 $$\begin{aligned} \gamma _{sep} = min\left\{ 2 max\left[ \left( \frac{Re_V}{3.235Re_{\theta c}} \right) -1, 0 \right] F_{reattach} , 2 \right\} F_{\theta t} \end{aligned}$$

The efficient intermittence factor $\gamma _{eff}$ that controls turbulent kinetic energy generation is:

5 $$\begin{aligned} \gamma _{eff}=max(\gamma ,\gamma _{sep}) \end{aligned}$$

For the transition momentum thickness Reynolds number $\widetilde{Re_{\theta t}}$, the transport equation is:

6 $$\begin{aligned} \frac{\partial \left( \rho \widetilde{Re_{\theta t}} \right) }{\partial t} + \frac{\partial (\rho U_j \widetilde{Re_{\theta t}})}{\partial x_j} = P_{\theta t} + \frac{\partial }{\partial x_j}\left[ \sigma _{\theta t} (\mu + \mu _t) \frac{\partial \widetilde{Re_{\theta t}}}{\partial x_j} \right] \end{aligned}$$

### Geometry and boundary conditions

**Figure 1:**
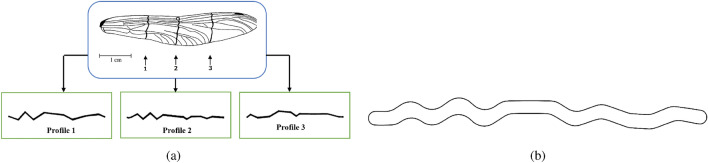
(**a**) Three cross-sections of a dragonfly forewing (Aeshna Cyanea)^37,38^ (**b**) Final cross section profile based on profile 2^39^.

The corrugated and NACA4412 airfoils were selected to be investigated the ground effect on aerodynamic coefficients in flapping motion. The corrugated airfoil implemented in the present simulation corresponds to the cross-section of a dragonfly forewing (Aeshna Cyanea). Along the dragonfly’s wing span, Kesel extracted three cross-sections^37,38^. As illustrated in  Figure 1a, these sections were labeled profile numbers 1, 2, and 3. So, the geometry of the dragonfly’s wing profile varies depending on where you are on the span. Profile number two was chosen for this study.  Figure 1b depicts the final section after the sharp edges of the profile were smoothed off^39^. As a result of this change, the corrugated airfoil’s overall geometry is unaffected.

**Figure 2:**
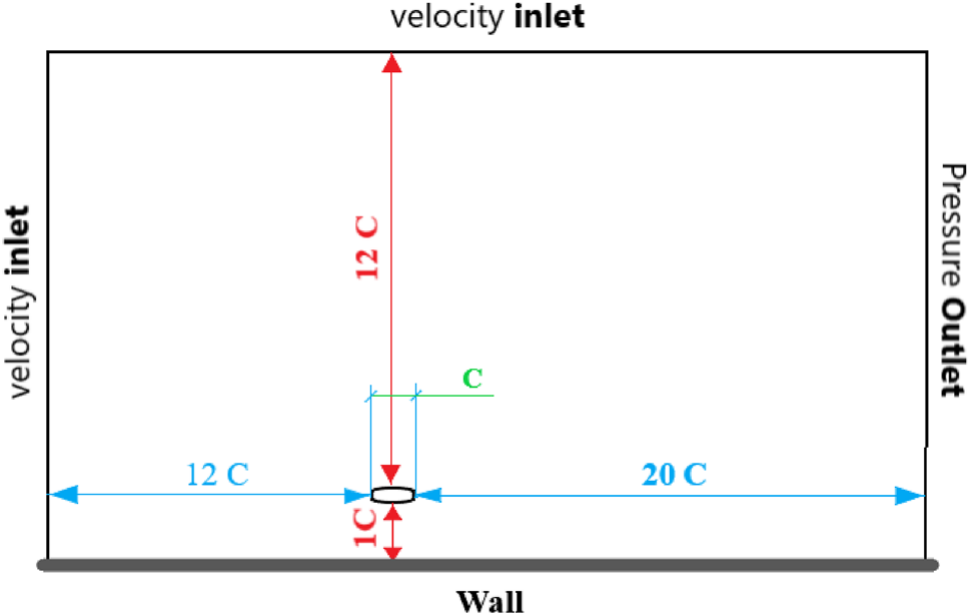
Computational domain and applied boundary conditions around airfoil.

The computational domain is rectangular with a circular subdomain. The distance between the center of the airfoil, inlet, outlet, and the upper boundary is 12c, 20c, and 12c ( Fig. 2). For the flow simulation, three boundary conditions were used. The velocity at the inlet and upper boundaries were units, the no-slip condition was applied along the wall, and zero gradient conditions were implemented at the outlet boundary. Based on inlet flow velocity and airfoil chord, the *Re* was set to $5\times 10^4$.

**Figure 3:**
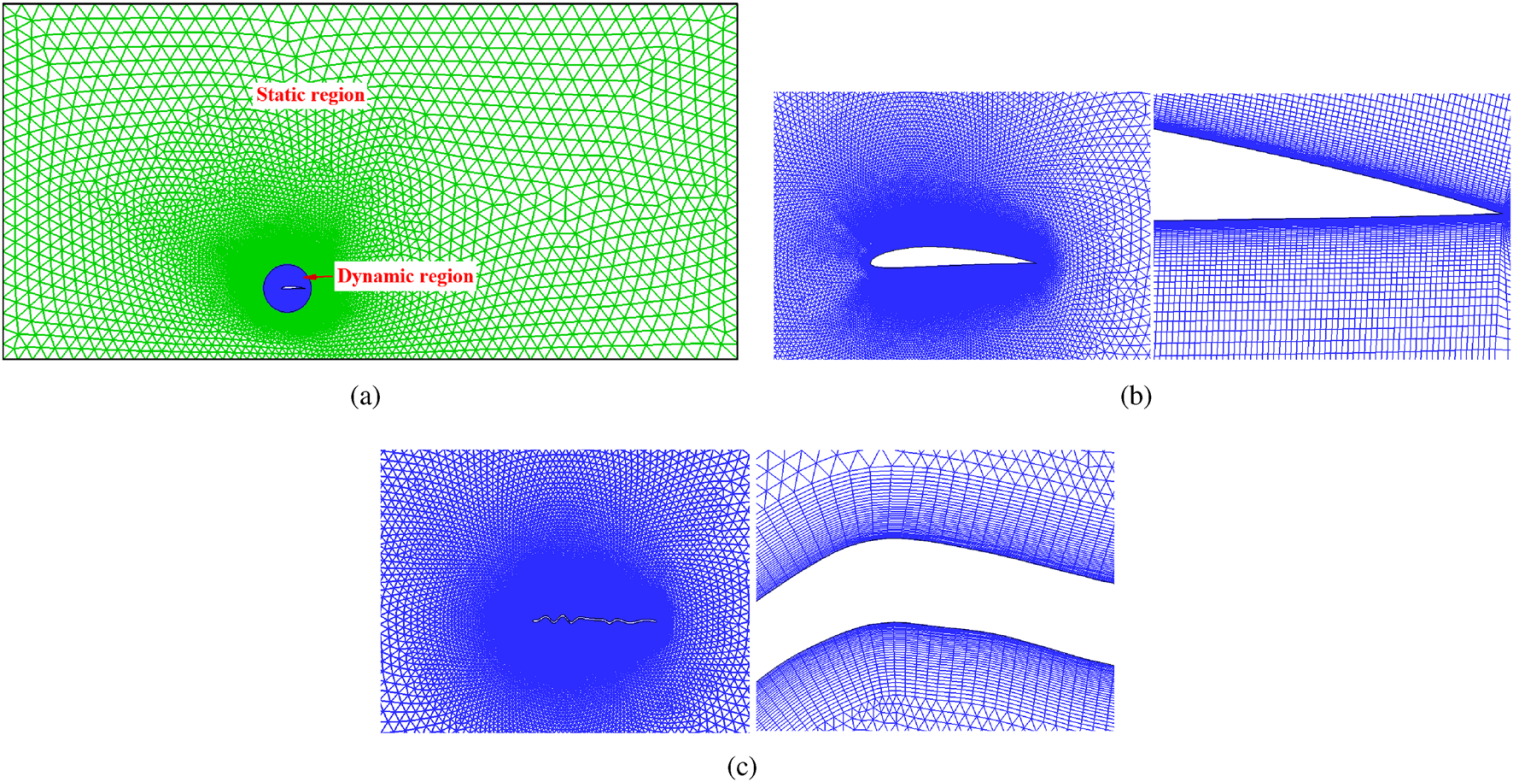
(**a**) Unstructured grids around airfoils (**b**) Close-up view of grid near NACA 4412 airfoil (**c**) Close-up view of grid near dragonfly airfoil.

The simulation domain is divided into two regions, as shown in  Fig. 3. The first region is the circle surface which is the rotating part of the domain called “Dynamic Region”. The second region is static region that is considered to be far-field. Furthermore, hybrid mesh type is used for this research. As depicted in  Fig. 3, c-type grid is utilized in the vicinity of the airfoil and the rest is unstructured tri-elements.

### Grid and time step independence study

**Figure 4:**
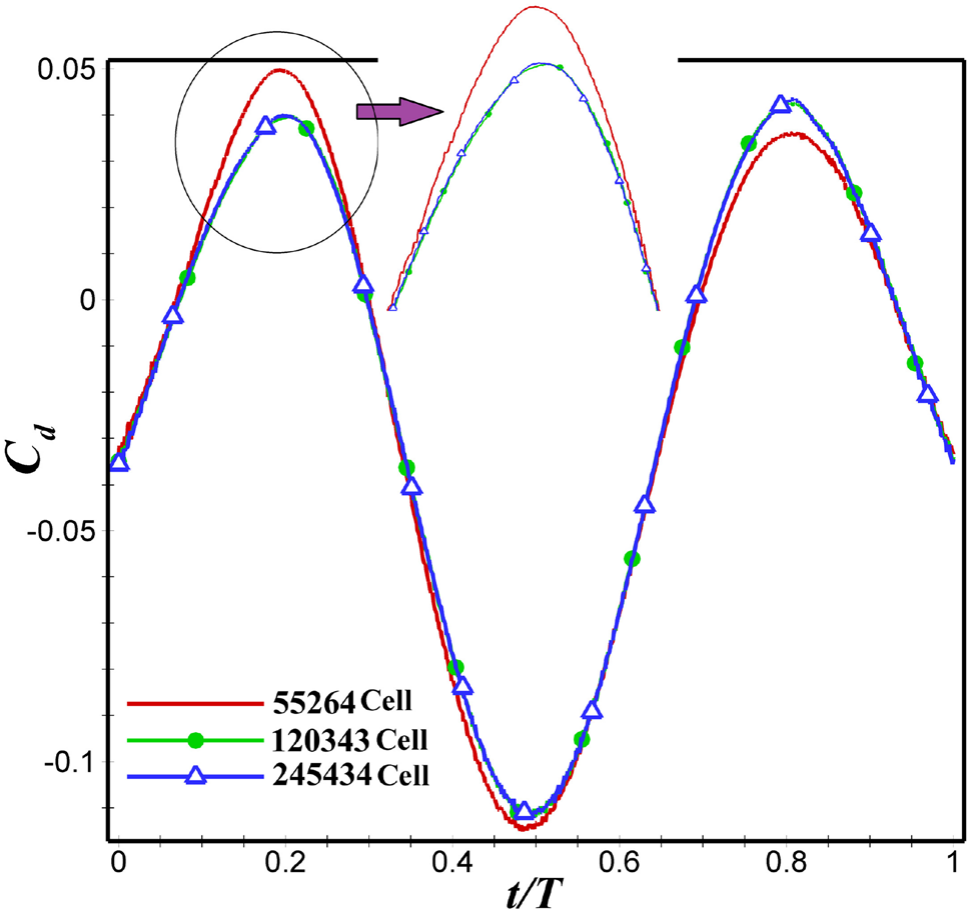
NACA 4412 airfoil drag coefficient variations for different grids with close-up views.

**Figure 5:**
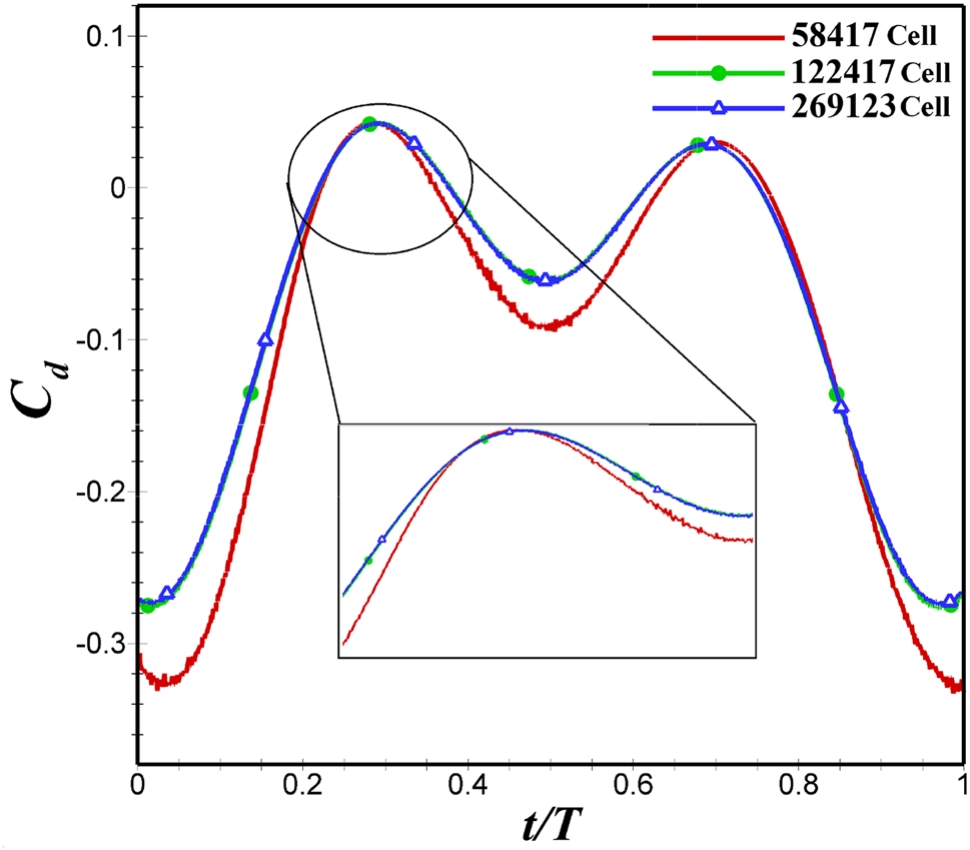
Dragonfly-inspired airfoil drag coefficient variations for different grids with close-up views.

In this section, three different number of cells (three coarse, medium, and fine grids) have been used to check the grid independence study for each airfoil. The number of nodes on both airfoil’s surface, in coarse, medium and fine grids is 200, 400, and 800, respectively. Further details can be found in  Table 2.  Figures 4 and  5 illustrates $C_d$ for NACA 4412 and dragonfly-inspired airfoils with different grids, respectively. As it can be observed that medium grid were not significantly different from the fine grid. As a result, the difference in $C_d$ between medium and fine grids is less than 2 percent. Therefore, for this study, medium grids with 120343 cells and 122417 cells were selected for NACA 4412 and dragonfly airfoils, respectively.

**Table 2:** Information of the computational grids.

Airfoils		Number of the node on the airfoil	Number of cells	Boundary layer		
				Number of layer	First layer height	Growth rate
NACA 4412	Coarse	200	55264	20	$6\times 10^{-5}$	1.2
	Medium	400	120343	40	$3\times 10^{-5}$	1.1
	Fine	800	245434	40	$3\times 10^{-5}$	1.1
Dragonfly	Coarse	200	58417	20	$6\times 10^{-5}$	1.2
	Medium	400	122417	40	$3\times 10^{-5}$	1.1
	Fine	800	269123	40	$3\times 10^{-5}$	1.1

**Figure 6:**
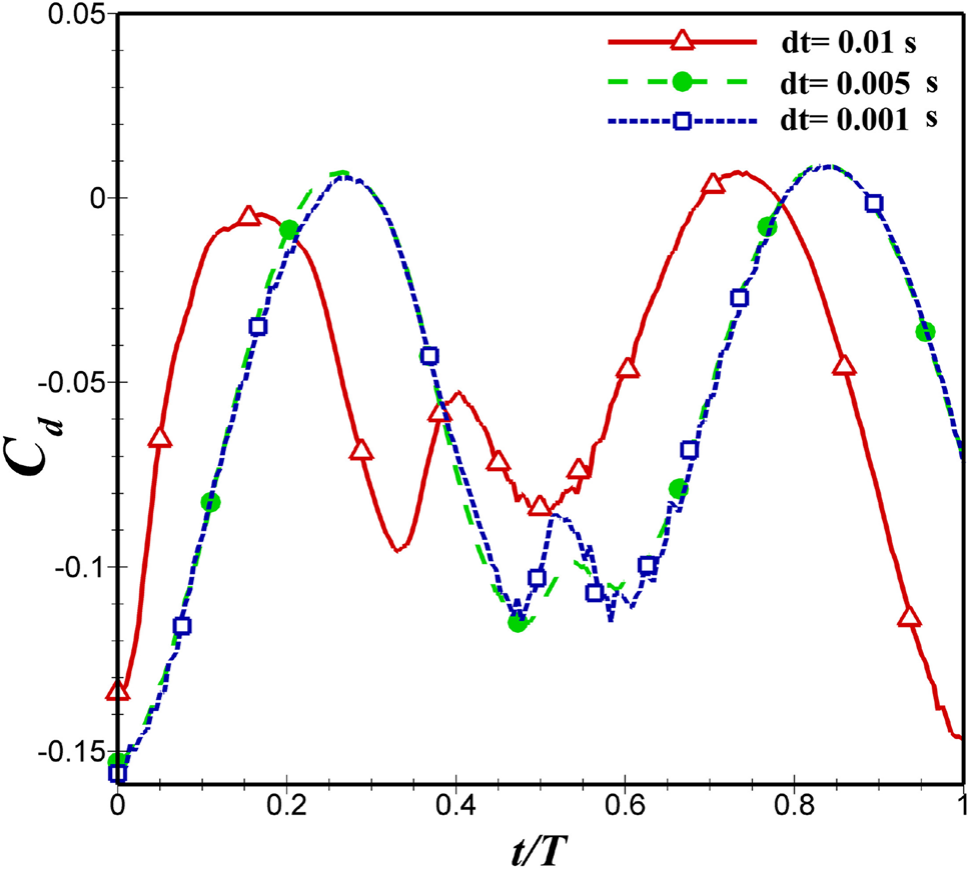
Drag coefficient variations versus time for different grids with close-up views.

The grid and time step sensitivity analysis is performed at pitching amplitude of 10 degrees, $St=0.4$ and $Re=5\times 10^4$. To investigate the sensitivity of time step independence, three different Time steps of 0.001, 0.005 and 0.01s have been chosen on the best grid for NACA 4412 airfoil. According to  Fig. 6, it can be said that $C_d$ between the time steps of 0.005s and 0.001s are very close together, which is due to this slight difference (about 1%) the time step 0.005 is chosen to save time of calculation.

### Numerical procedure

With an implicit scheme, an incompressible 2D finite volume CFD solver was employed for URANS. The turbulence model was implemented using the Transition SST model. The semi-implicit method used the pressure and velocity coupling used by the semi-implicit method for the pressure-linked equations (SIMPLE) algorithm. An iterative algorithm first guessed an initial pressure, and then momentum equations were solved. Afterward, a pressure equation for pressure correction was utilized to satisfy the continuity equation. Also, pressure, momentum, and turbulence transport equations are discretized using second-order spatial. Iterations are completed when all scaled residuals are less than $10^{-5}$. It is noted that the analysis was carried out on a computer with Intel®Core (TM) i7-7700 @ 3.6 GHz CPU and 32 GB RAM.

Furthermore, a non-steady numerical technique requires numerous timesteps to reach a solution. In this paper, we characterize a criterion for the convergence of solutions with the maximum values of lift and drag in every cycle; solutions are expected as converged if the difference between the maximum values of lift and drag in the present and the prior cycles are below 1% of the present cycle value. The convergence of the seventh cycle is identified. We implement the eighth cycle to ensure that the convergence of the solution is authentic; the results are positive. After that, we solely address the eighth cycle’s results.

### Solver validation

**Figure 7:**
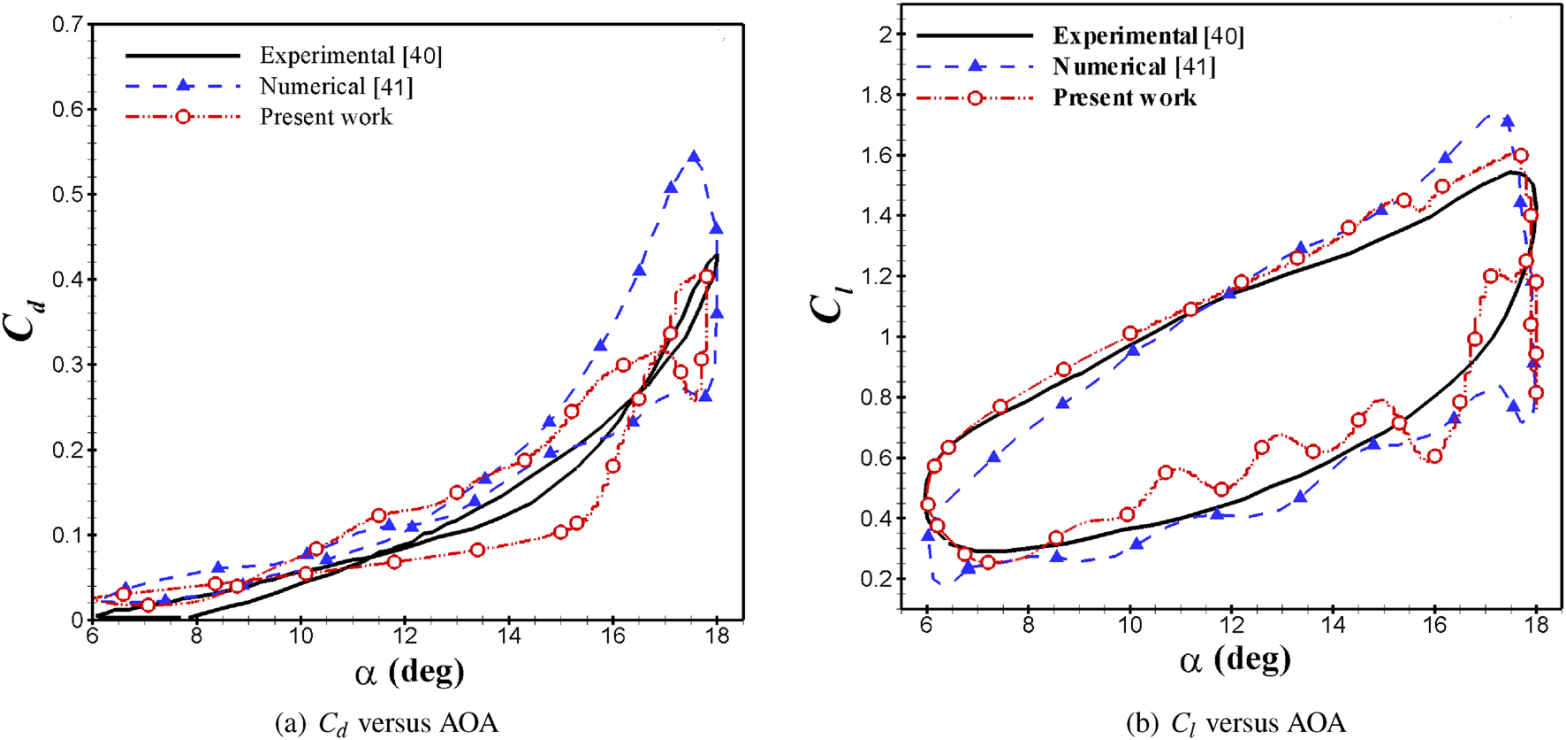
Comparison of aerodynamic coefficients with Refs.^40,41^.

In order to validate the solving method in this study, the results are compared with wind tunnel measurements^40^ and other URANS numerical^41^ test cases. It is a NACA0012 airfoil and moves with a pitching motion at a pitching amplitude of 6 degrees, frequency of 0.188, incidence angle of 12 degrees, and *Re* of $10^5$.  Figure 7 shows the comparison of aerodynamic coefficients with Refs.^40,41^. In terms of the lift coefficient, the new CFD results demonstrate better agreement with the experiment than earlier numerical studies in that the anticipated peak value is closer to the experimental observations. The projected peak value of the lift coefficient of the present work differs by 3% compared to the experimental measurements. While for Martinat et al.^41^, this difference is 11%. Also, the lift coefficient in the downstroke phase oscillates about the mean value acquired from the measurements. The projected peak value of the drag coefficient of the present work differs by 5% compared to the experimental measurements, while for Ref.^41^, this difference is 22%. Due to using the Transition SST model, current CFD outcomes project a secondary LEV that assists the recovery of lift and drag coefficients about the maximum angle of attack. Therefore, the accuracy of the present study is higher than Ref.^41^ and is closer to the experimental results.

### Motion equation

**Figure 8:**
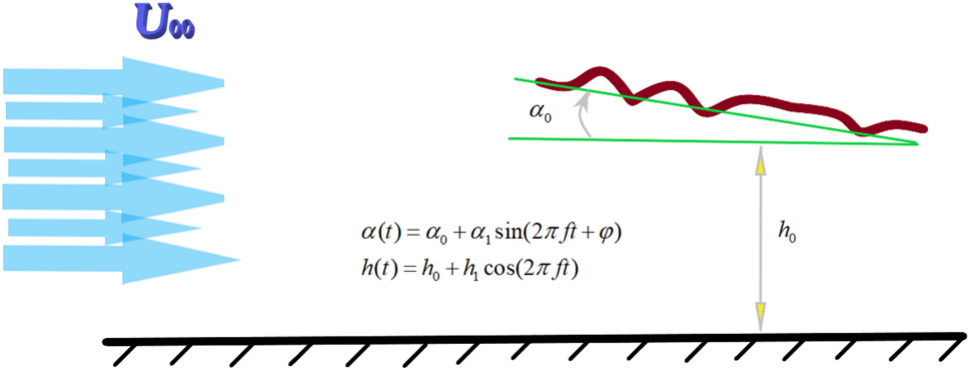
Flow over a flapping airfoil in ground effect.

The flapping airfoil’s motion involves both pitching and plunging motions. As shown in  Fig. 8, the airfoil movement is expressed by Eqs. (7) and  (8).

7 $$\begin{aligned} \alpha (t)= & {} \alpha _0 + \alpha _1 \sin (2\pi ft+\phi ) \end{aligned}$$

8 $$\begin{aligned} h(t)= & {} h_0 + h_1 \cos (2\pi ft+\phi ) \end{aligned}$$

where $\alpha _1$ and $h_1$ represent the pitching and plunging amplitudes, respectively; $\alpha _0$ is the incidence angle of the airfoil; $h_0$ is the initial distance from the ground; *f* is the oscillation frequency, and is the phase difference between the pitching and plunging motions. The Strouhal number of oscillations can be defined as $St=kh_1/\pi c$, where c is length of airfoil chord and *k* is the reduced frequency. Reduced frequency is the dimensionless number used in general for the case of unsteady aerodynamics. Reduced frequency is given by the expression $k=\omega c/U_{\infty }$, $\omega =2\pi f$ and U are circular frequency and free stream velocity, respectively. The parameters selected for the current study are presented in  Table 3.

**Table 3:** Selective parameters for present study.

Parameters	Value
*Re*	$5\times 10^3$ & $5\times 10^4$
$\alpha _0$	0
$\alpha _1$	10
$h_1$	0.4c
$\phi$	0
$h_0$	$c\le h_{0}\le 5c$
*St*	$0.2\le St\le 0.6$

## Results and discussion

This section investigates the impact of the ground effect on the aerodynamic performance of dragonfly and NACA4412 airfoils. Moreover, the impact of mean ground clearance, the St, and Re are discussed.

### Effect of the mean distant from the ground

**Figure 9:**
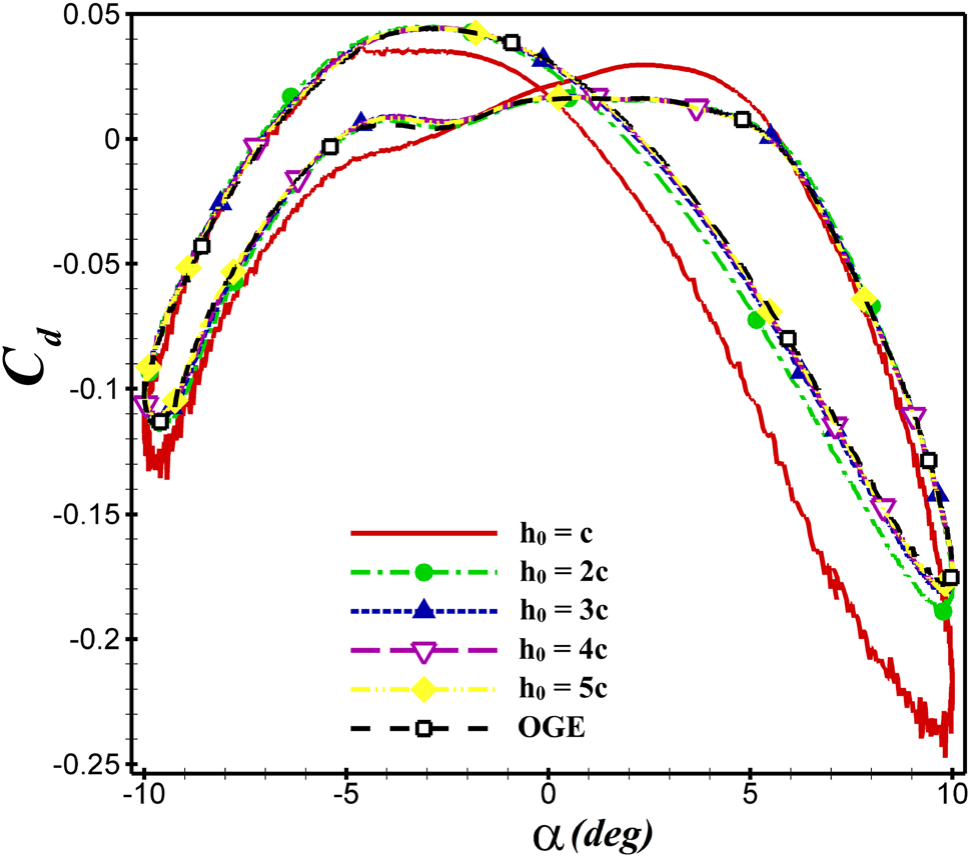
The $C_d$ of dragonfly airfoil in different $h_0$ over one oscillation period (OGE means out of ground effect).

**Figure 10:**
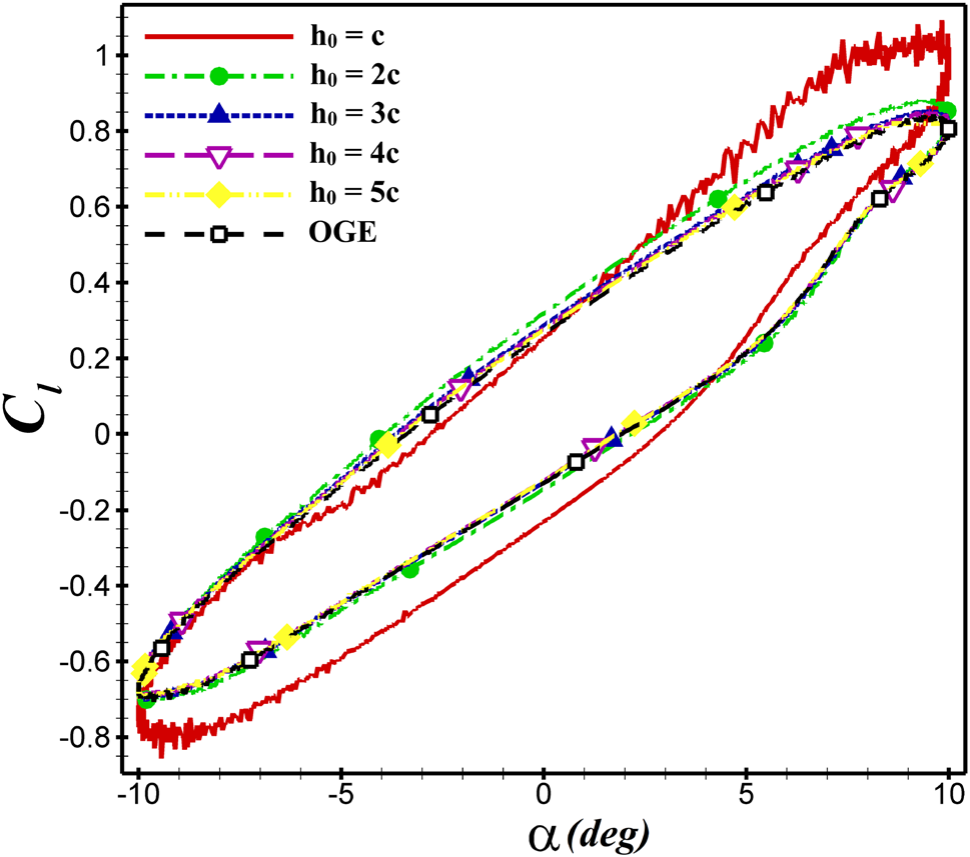
The $C_l$ of dragonfly airfoil in different $h_0$ over one oscillation period.

The *Re* and *St* are fixed at $5\times 10^4$ and 0.4, respectively. The airfoil’s mean distance from the ground is varied from c to 5c ($5c\le h_{0}\le c$). In  Fig. 9, the time history of $C_d$ for dragonfly airfoil is shown at various distances from the ground. As the ground level approaches, the hysteresis loop becomes wider, and the value of thrust (negative $C_d$) produced increases. As illustrated in  Fig. 10, when $h_0$ is changed from 5c to 2c, the values of the average lift coefficient over one oscillation period ($\overline{C_l}$) increase by approximately 1.1%. However, when the distance is reduced from 2c to c, the $\overline{C_l}$ increases from 0.085 to 0.095 (11.7%), which is significant. Based on this figure, a reduction in $h_0$ increases $C_l$.

**Figure 11:**
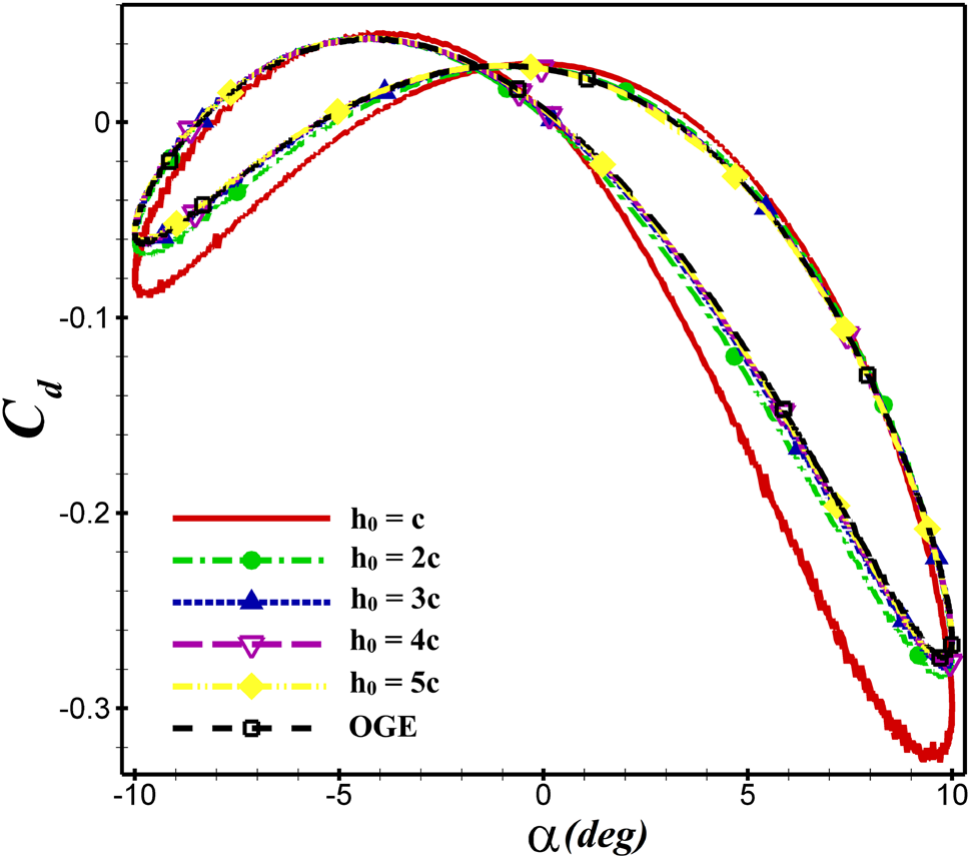
The $C_d$ of NACA 4412 airfoil in different $h_0$ over one oscillation period.

**Figure 12:**
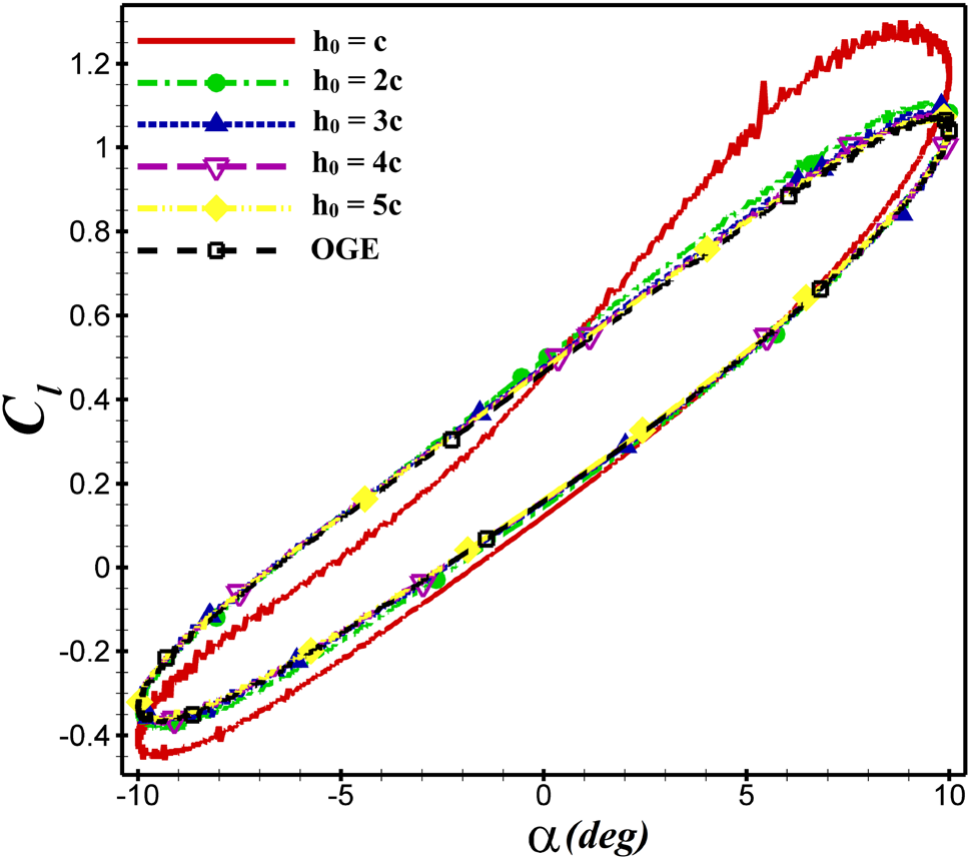
The $C_l$ of NACA 4412 airfoil in different $h_0$ over one oscillation period.

Similarly, the average thrust and lift coefficients of NACA4412 airfoil at $h_0$= c than 2c increases by 35% and 15.5%, respectively as seen in  Figs. 11 and 12. For both airfoils, $\overline{C_d}$ and $\overline{C_l}$ values are shown in  Table 4. The aerodynamic coefficients do not change significantly, when the mean ground clearance alters from 5c to c. But if, it is equal to $h_0$ =c, the aerodynamic coefficients are greatly affected, and the ground effect is very apparent.

**Table 4:** The values of the average drag and lift coefficients over one oscillation period at various $h_0$.

The mean ground clearance	Dragonfly airfoil		NACA 4412 airfoil	
	$\overline{C_d}$	$\overline{C_l}$	$\overline{C_d}$	$\overline{C_l}$
$h_0$=c	− 0.0704	0.095	− 0.112	0.403
$h_0$=2c	− 0.0543	0.0854	− 0.0828	0.349
$h_0$=3c	− 0.0531	0.0844	− 0.0738	0.339
$h_0$=4c	− 0.0538	0.0838	− 0.0727	0.341
$h_0$=5c	− 0.0573	0.0834	− 0.0713	0.339
OGE	− 0.0505	0.0831	− 0.0696	0.326

**Figure 13:**
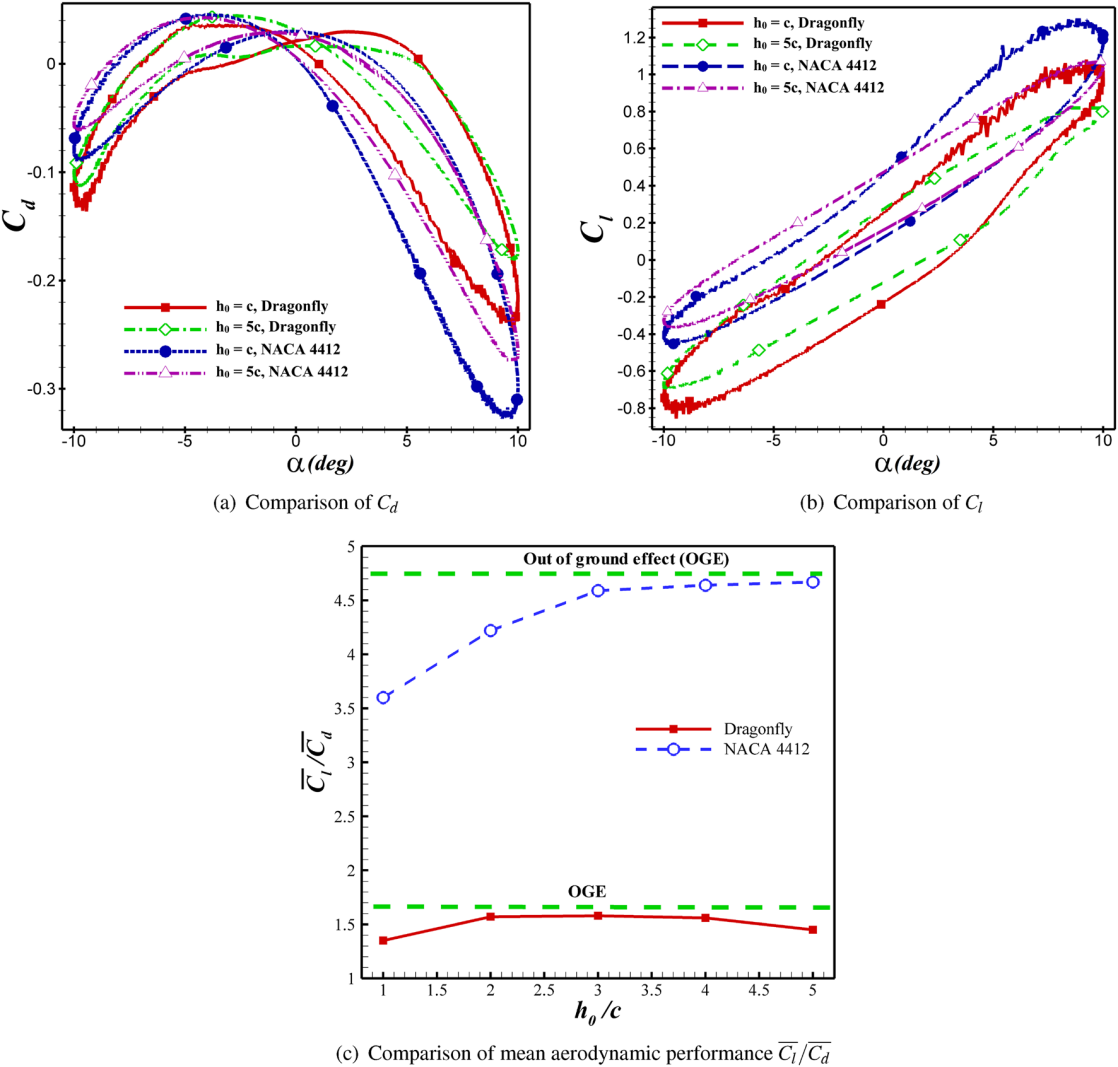
Comparison of $C_d$, $C_l$ and $\overline{C_l}/\overline{C_d}$ between dragonfly and NACA 4412 airfoils in different distant from the ground at $St=0.4$ and $Re= 5\times 10^4$.

For the NACA 4412 and dragonfly airfoils, the $C_d$ and $C_l$ coefficients are compared at $h_0$=5c and $h_0$=c in  Fig. 13. By changing $h_0$=5c to $h_0$=c, the values of $\overline{C_d}$ decreased from −0.0713 to −0.112 and −0.0573 to −0.0704 for NACA 4412 and dragonfly airfoils, respectively. As shown in  Fig. 13a, the hysteresis loop of $C_d$ is lower for the dragonfly airfoil compared to NACA 4412 airfoil at AoA<0, which indicates that the dragonfly airfoil provides greater thrust than NACA 4412. In contrast, for AOA>0, the $C_d$ hysteresis loop of dragonfly airfoil is higher, so the $C_d$ of NACA 4412 airfoil is lower. This situation is valid for all examined cases. The mean thrust coefficient of NACA 4412 airfoil is 24.4% higher than that of dragonfly airfoil at $h_0$=5c, whereas this value reaches 59% at $h_0$=c. When ground clearance alters from $h_0$=5c to $h_0$=c, the $\overline{C_l}$ value of NACA 4412 and dragonfly airfoils augments from 0.339 to 0.403 and from 0.0834 to 0.095, respectively. The change of $C_l$ depending on the $h_0$ is illustrated in  Fig. 13b for various AoA. As the hysteresis loop of $C_l$ for NACA 4412 airfoil is higher than that of dragonfly airfoil at $h_0$=c and $h_0$=5c, it has a higher lift coefficient. At h=c, $C_l$ of NACA 4412 is significantly higher than dragonfly airfoil. The most effective ground clearance on the aerodynamic performance of the both airfoils is the $h_0$=c as it results in a higher $C_l$ and lower $C_d$ than the other cases.

The mean aerodynamic performance ($\overline{C_l}/\overline{C_d}$) behavior of the flapping airfoils close to the ground is shown in Fig.11. The dashed line represents the results of out-of-ground (OGE) or $h_0/c = \infty$. From the figure, it is found that the curves of $\overline{C_l}/\overline{C_d}$ become steeper and steeper when $h_0$ decreases. It means that the ground effect gets stronger as the airfoil gets closer and closer to the ground. According to  Fig. 13c, the presence of the wall can diminish $\overline{C_l}/\overline{C_d}$ clearly. The minimum value of $\overline{C_l}/\overline{C_d}$ occurs at $h_0/c = 1$. The aerodynamic performance of both airfoils increases with enhanced distance from the ground until reaching the value of the state OGE.

### Effect of Strouhal number

**Figure 14:**
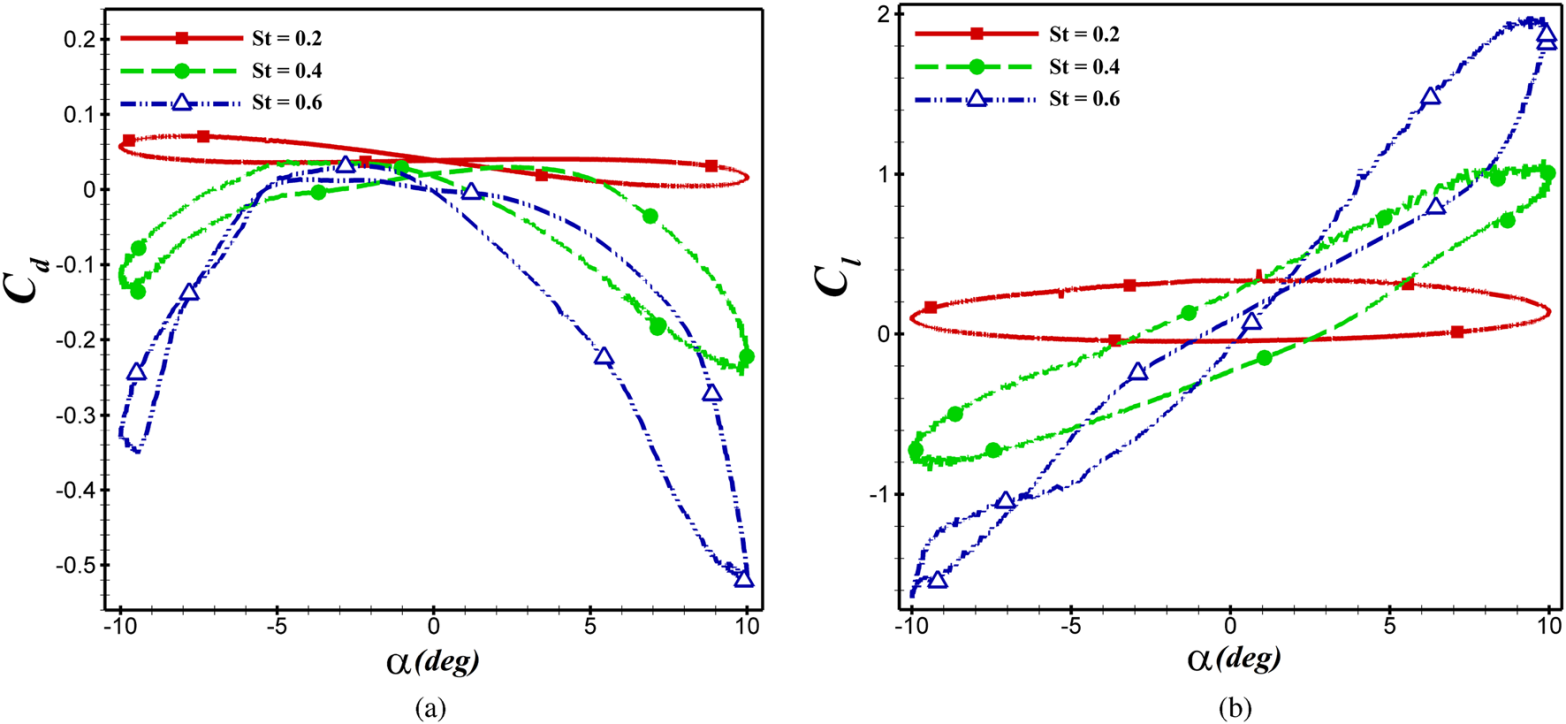
The $C_d$ and $C_l$ of dragonfly airfoil in different *St* at $Re= 5\times 10^4$.

**Figure 15:**
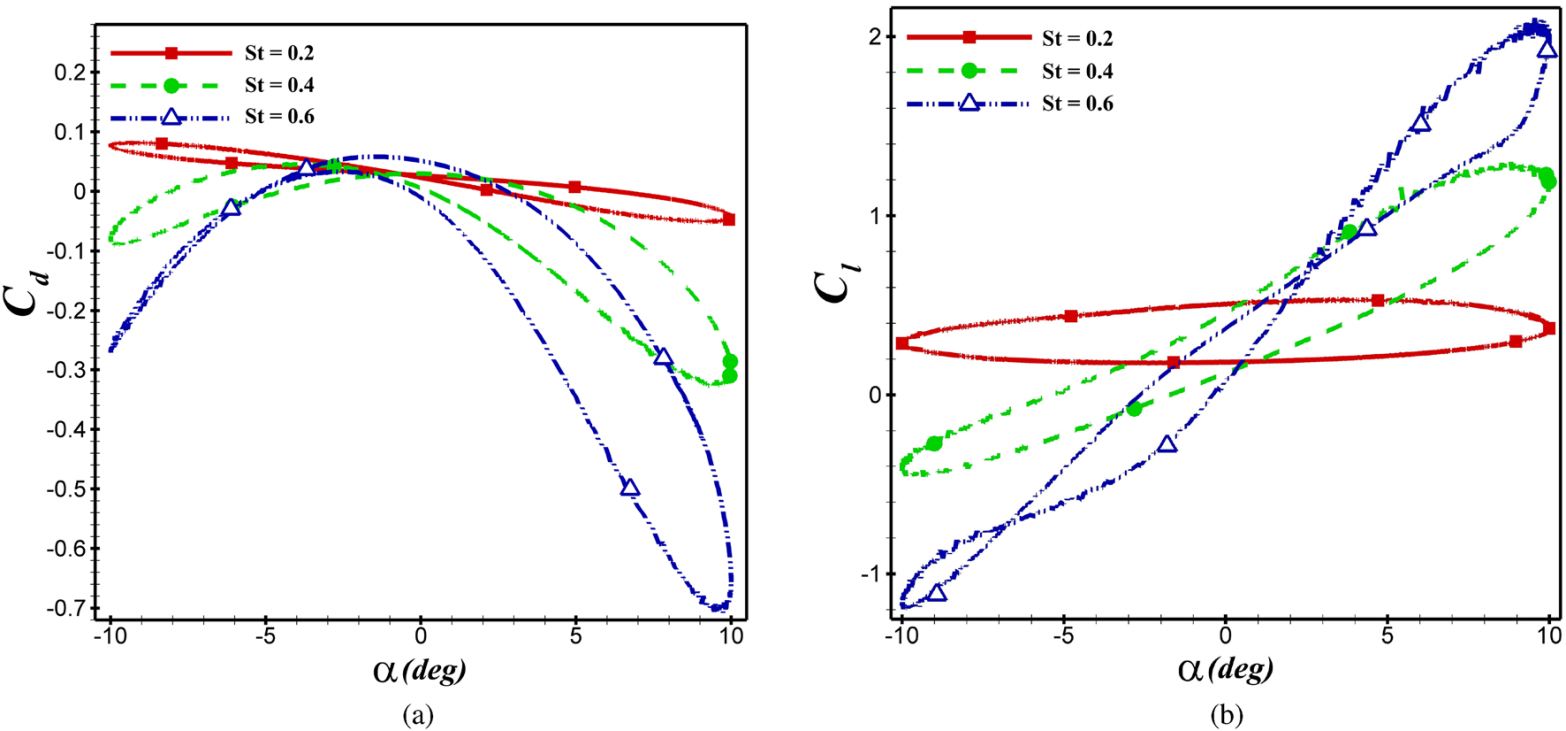
The $C_d$ and $C_l$ of NACA 4412 airfoil in different *St* at $Re= 5\times 10^4$.

In this section, the effect of the Strouhal number on aerodynamic coefficients is investigated by fixing the $Re=5\times 10^4$ and $h_0$=c. The impact of *St* on aerodynamic coefficients of the NACA 4412 and dragonfly airfoils at various AoA’s are demonstrated in  Figs. 14 and  15, respectively. Based on these figures, it can conclude that an increase in *St* results in a decrease in $C_d$ (or augments thrust) and an increase in $C_l$. Moreover, the slope of the hysteresis loop increment by increasing *St*, as inferred from the Figures. In other words, by increasing *St*, the $C_l$ increases at the same AoA.

The aerodynamic coefficients of both airfoils are presented at various *St* in  Table 5. For instance, for a dragonfly airfoil, the $\overline{C_l}$ increases from 0.127 to 0.218, and the $C_d$ decreases from 0.0394 to -0.202 when *St* alters from 0.2 to 0.6.

**Table 5:** The mean aerodynamic coefficients of both airfoils at various *St*.

Parameter	Dragonfly airfoil		NACA 4412 airfoil	
	$\overline{C_d}$	$\overline{C_l}$	$\overline{C_d}$	$\overline{C_l}$
*St*=0.2	0.0394	0.127	0.0176	0.346
*St*=0.4	−0.0721	0.095	−0.112	0.403
*St*=0.6	−0.202	0.218	−0.226	0.472

**Figure 16:**
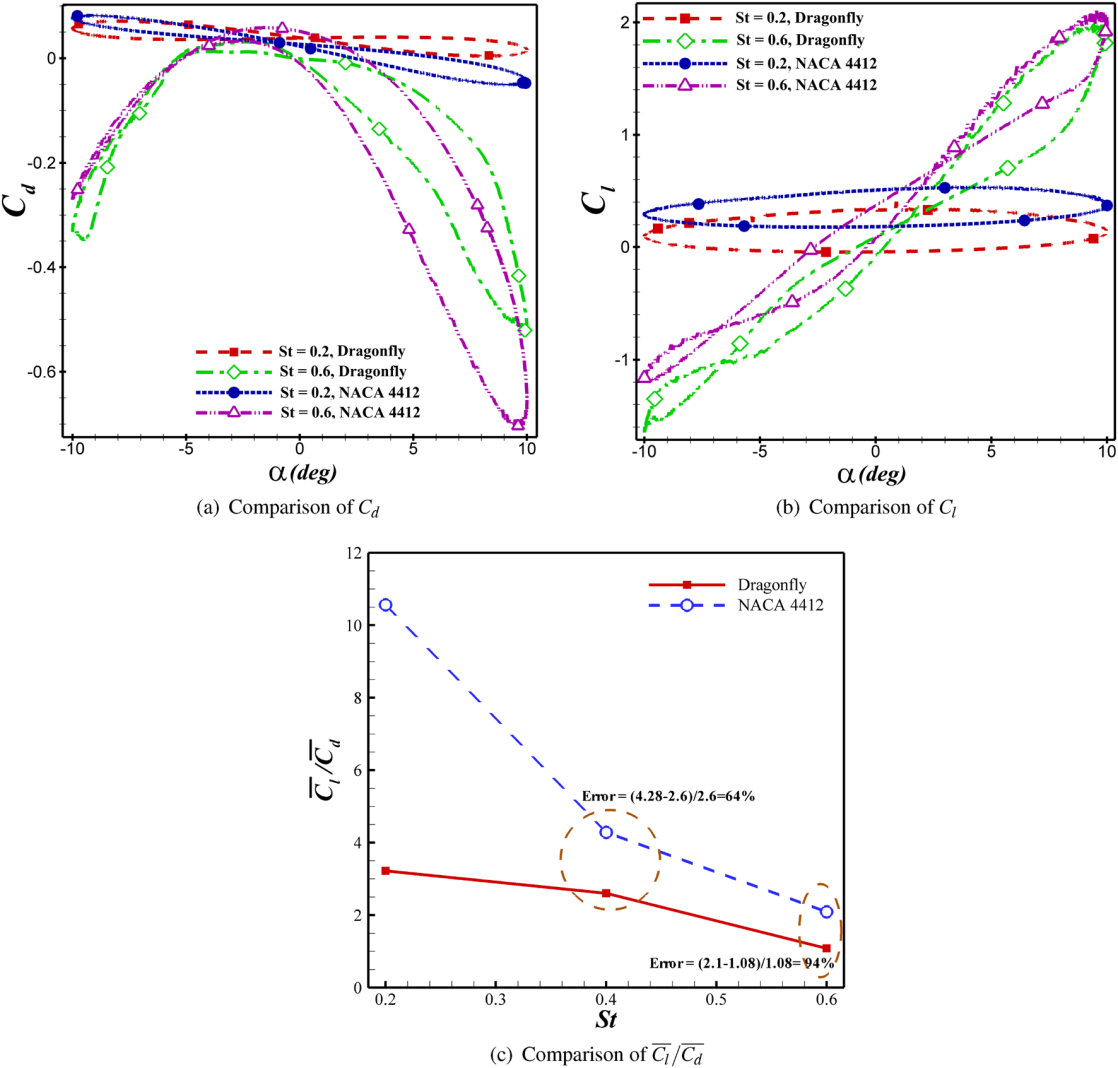
Comparison of $C_d$, $C_l$, and $\overline{C_l}/\overline{C_d}$ between dragonfly and NACA 4412 airfoils in different *St* at $Re= 5\times 10^4$.

In  Fig. 16, the $C_d$ and $C_l$ are compared at *St*=0.2 and 0.6 for the NACA4412 and dragonfly airfoils. As can be seen in  Fig. 16a, at AoA<0, the value of $C_d$ for the dragonfly airfoil is lower, whereas, at AoA>0, there is a lower value of $C_d$ for the NACA4412 airfoil. This situation is valid for *St*=0.2 and 0.6.  Fig. 16b demonstrates the $C_l$ hysteresis loop for the NACA4412 airfoil is higher than the dragonfly airfoil, and consequently has a higher lift coefficient. This situation is valid for *St*=0.2 and 0.6. The $C_d$ of the NACA4412 airfoil is 55% and 12% lower than that of dragonfly airfoil at *St*=0.2 and 0.6, respectively, and its $C_l$ is 63% and 54% higher. Therefore, NACA4412 airfoil has better aerodynamic performance (See  Fig. 16c).

**Figure 17:**
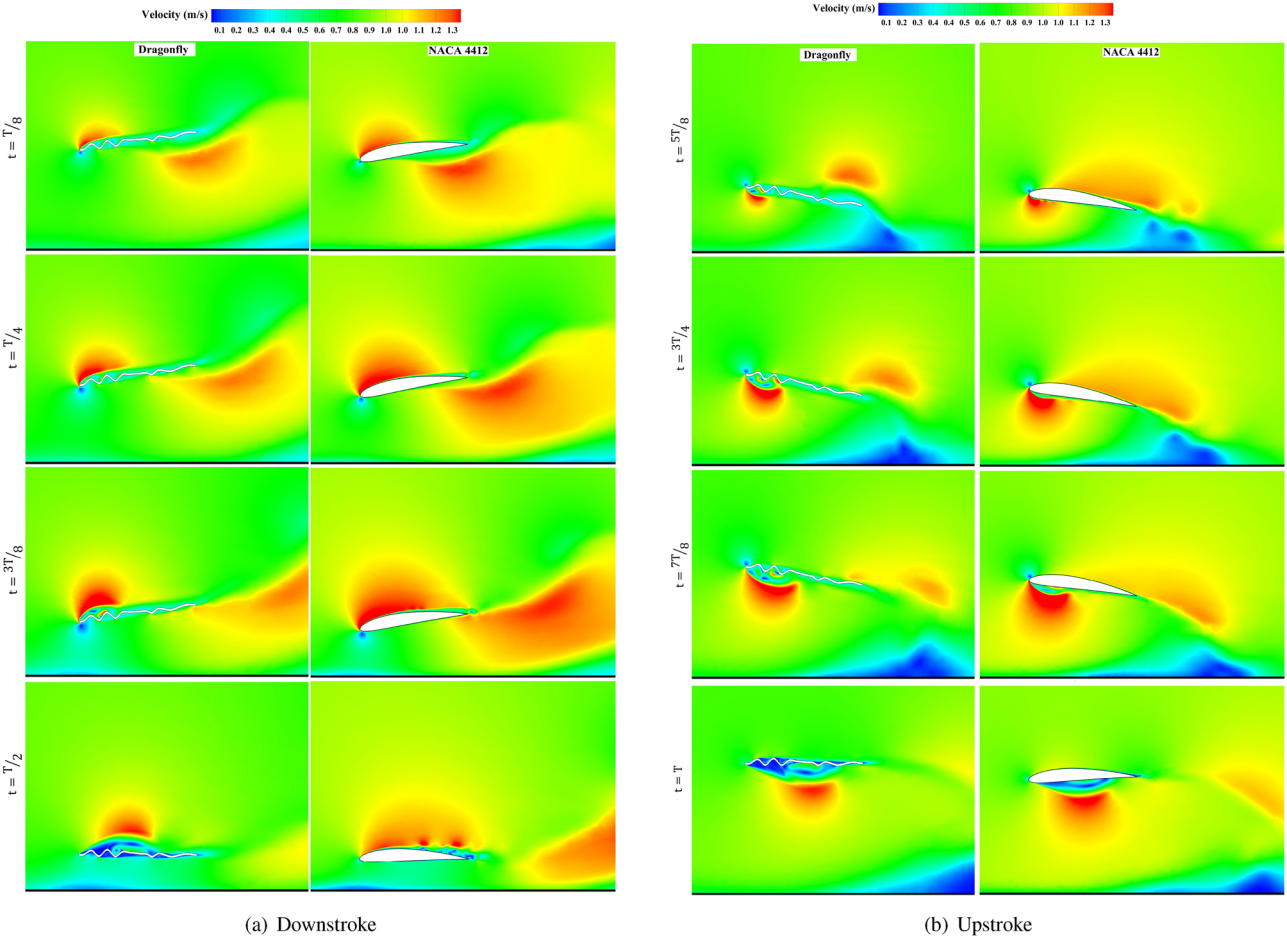
Contours of momentary velocity coefficients across a cycle with $Re= 5\times 10^4$ and *St*=0.6 for both dragonfly and NACA4412 airfoils.

Figure 17 illustrates the instantaneous velocity contours for the eight distinct time instants. Since the airfoils reach a quarter of the cycle period, they enter the dynamic stall region. The leading edge vortex starts moving towards the trailing edge and separates from the airfoil. Simultaneously, as new LEVs are generated, the flow is reattached; then, the lift force is recovered. Therefore, this phenomenon will bring many fluctuations in the lift plot. When the airfoil continues downstroke, and the airfoil and ground surface distance decrease, the low-pressure zone surrounding the lower surface of the airfoil moves to the trailing edge and becomes weaker. Hence, the LEV is intensified to some extent as the airfoil approaches the end of the downstroke at $t/T=1/2$ (exactly a bit of an instant before the end of the downstroke). Consequently, the airfoil’s upper surface pressure is greatly decreased, forming a suction zone, the source of the positive lift force development.

As a consequence of the ground effect, a greater pressure zone generated at the bottom surface of the airfoil acts like a trampoline. The LEV proceeds over the airfoil’s upper surface and begins to disengage from the airfoil tail when the airfoil reverses its motion direction at *t*/*T*=1/2. A low-pressure zone is again produced below the leading edge to produce LEV; then, the vortex interacts with the ground, slowing LEV separation and improving lift force increase.

The obvious difference between the two geometries introduced at $Re=5\times 10^4$ is the trapping of airflow between the peaks and valleys of the pleated airfoil and the creation of a very low-velocity region - almost zero - which is the main advantage of bio airfoils over those of conventional. Since the fluid flow moves on the coiled fluid flow in the folds of the bio airfoil, it greatly reduces skin friction.

The next point in the figure of the speed contour is the lower speed gradient in the dragonfly airfoil compared to NACA4412, leading to the formation of vortices on the leading and trailing edges with a greater distance from each other on the dragonfly airfoil in comparison with the NACA4412. This event helps to increase the speed of rotation around the airfoil; however, due to the high flow momentum and the thickness of the dragonfly airfoil at $Re=5\times 10^4$, the lift force for the dragonfly airfoil is less than the cambered airfoil of NACA4412.

### Effect of Reynolds number on aerodynamic performance

**Figure 18:**
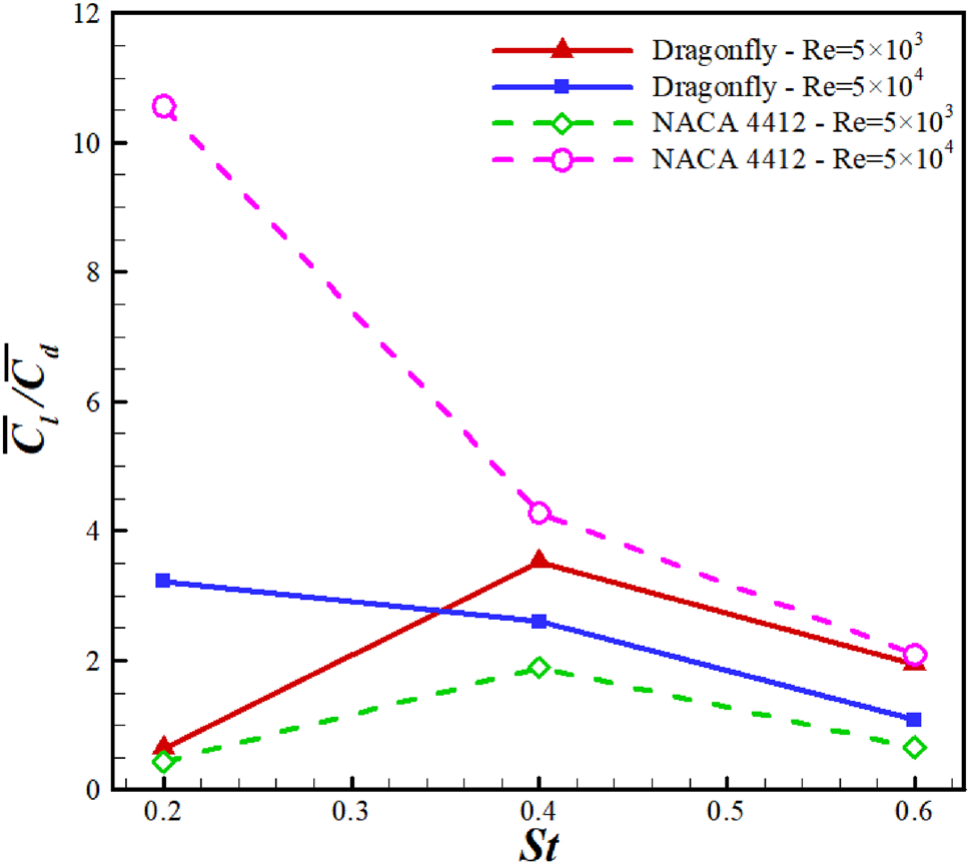
The mean aerodynamic performance variations in different *St*.

For the NACA4412 and dragonfly airfoils, the $C_l$/$C_d$ is compared at $Re=5\times 10^4$ and $Re=5\times 10^3$ in  Fig. 18. At $Re=5\times 10^4$, the values of $C_l$/$C_d$ decrease from 10.34 to 2.1 for NACA4412 airfoil and 3.22 to 1.8 dragonfly airfoil when St alters from 0.2 to 0.6. The efficiency difference between the two airfoils at *St*=0.2, 0.4, and 0.6 is approximately 211%, 64%, and 94%, indicating that the $C_l$/$C_d$ difference decreased substantially with increasing frequency. As a result, $C_l$/$C_d$ of NACA4412 airfoil is better than that of dragonfly airfoil, especially at *St*=0.2. For $Re=5\times 10^3$, it was found that dragonfly airfoil was better $C_l$/$C_d$ in all frequencies than NACA4412 airfoil. At *St*= 0.4, both airfoils exhibit maximal aerodynamic performance.

**Figure 19:**
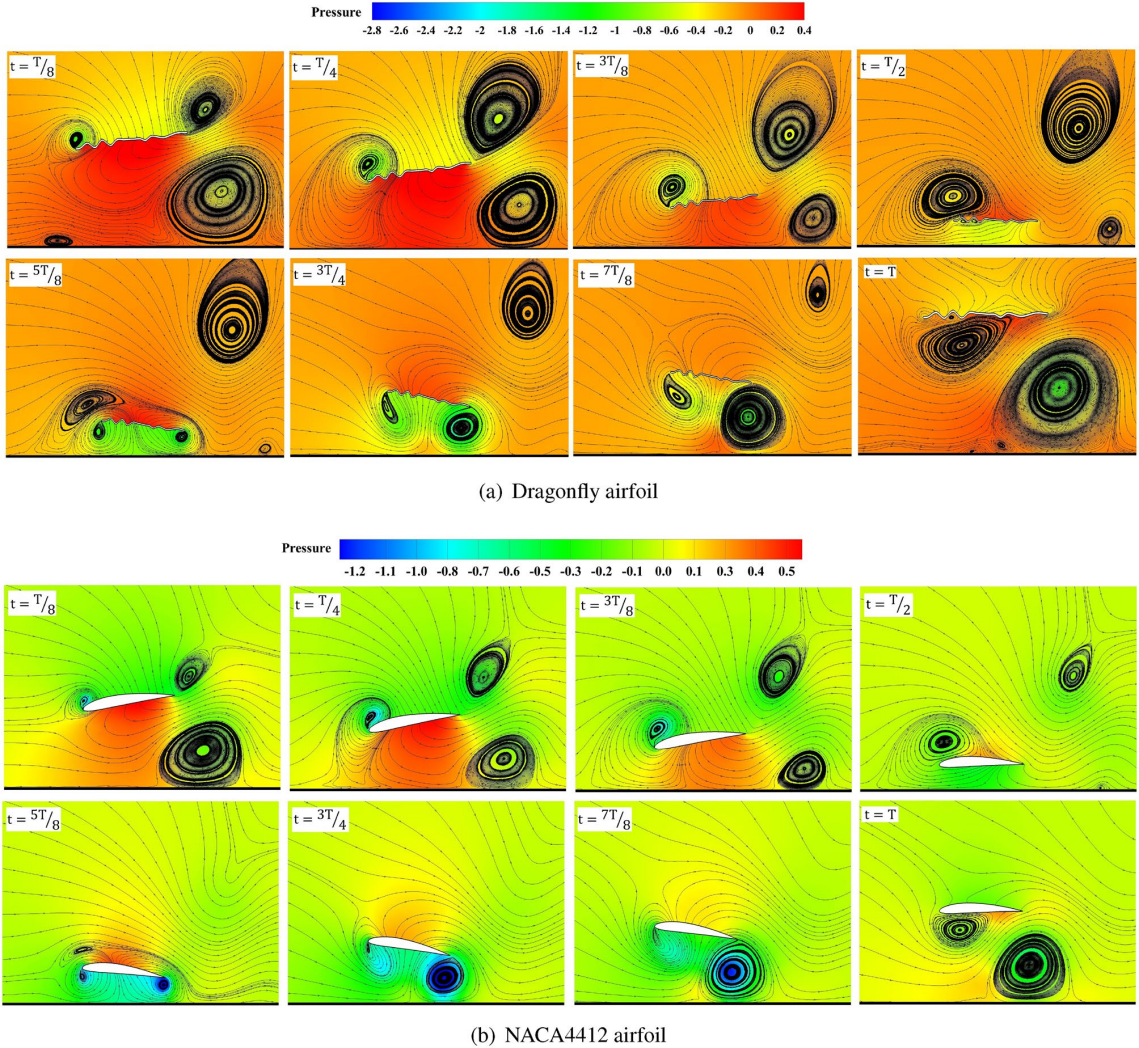
Contours of momentary pressure coefficients across a cycle with streamlines at $Re= 5\times 10^3$ and $St= 0.6$ for (**a**) NACA4412 and (**b**) dragonfly airfoils.

In  Fig. 19 , the airfoil begins to rotate counterclockwise, and as the airfoil advances toward the ground, the low-pressure zone formed under the airfoil starts to weaken. Accordingly, the vortex created at the ground surface is shrunk, and the separated vortices from the trailing edge move upwards as the low-pressure area under the airfoil is weakened. At *t*/*T*=5/8, at the end of the downstroke, two low-pressure zones are strengthened on the leading and trailing edges. As flapping proceeded, changing the clockwise rotation of the airfoil trailing edge vortex separated. By moving away from the ground surface, a vortex is formed under the airfoil, and the high-pressure zone on the upper airfoil surface is weakened.

The cushioning effect is a momentary rise in lift and drop in drag induced by air compression between the airfoil and the surface below while flight near the ground or water. The rising pressure on the airfoil’s bottom surface increases Cl. Along with the cushion effect, the delayed stall mechanism creates an LEV that results in a low-pressure zone on the airfoil’s top surface. A counterclockwise (CCW) trailing edge vortex (TEV) is also made close to the airfoil trailing edge. The increased Cl found in the near-ground situations is due to LEV combined with the cushion effect.

The flow made by the airfoil produces shear layers on the ground. As the airfoil begins to pitch up, the LEV and TEV of the airfoil are shed, leading to a lift force loss. At *t*/*T*=1/2, the airfoil interacts with and destroys the ground shear layer. The Cl becomes minimal due to the strong LEV generated beneath the airfoil and zero AoA. The airfoil begins its upstroke at *t*/*T*=5/8, and the lift gradually increases due to the caused velocity of the jet made by the shedding LEV and TEV. The LEV shed at the end of the downstroke collides with the ground, forming a new rebound vortex. The shift in the effective AoA of the airfoil caused by the flow due to the CW rebound vortex is a significant detection in this study. Whenever the airfoil is on its downstroke, the circulation of the rebound vortex to the surrounding fluid affects the AoA of the airfoil. Depending on the size and strength of the CW rebound vortex, the airfoil’s vertical force can increase or decrease.

**Figure 20:**
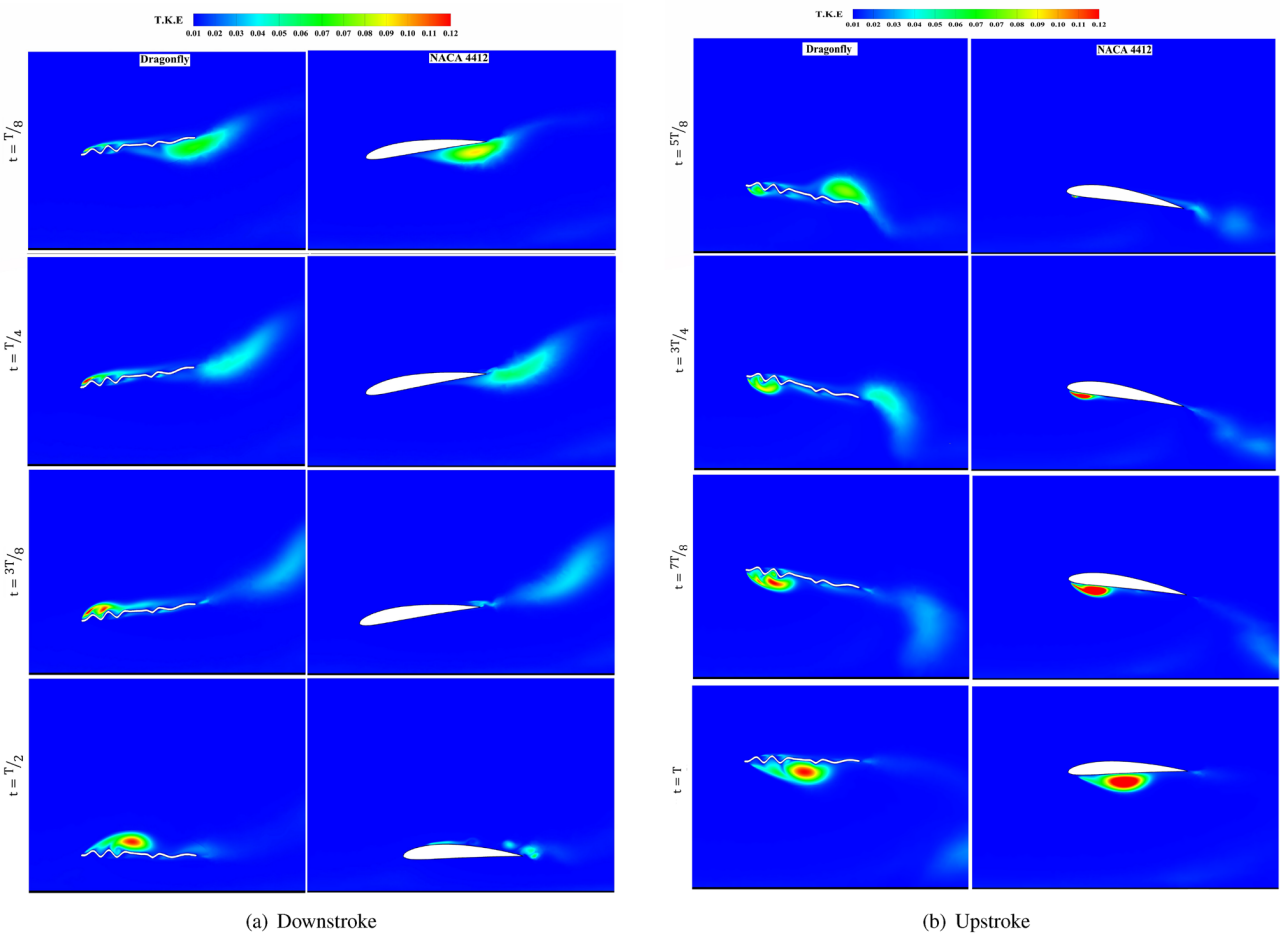
Contours of momentary turbulent kinematic energy (T.K.E) across a cycle with $Re= 5\times 10^4$ and $St= 0.6$ for both dragonfly and NACA4412 airfoils.

As illustrated in  Fig. 20, the dragonfly airfoil creates concentrated and stronger vortices than the NACA 4412 airfoil due to its passive flow control. However, because of the high velocity of the far-field flow, the trapped eddy in the corrugation cannot hold the vortices near the surface of the airfoil. As a result, a decrease in dragonfly-inspired airfoil performance is observed at high Re. As turbulent vortices rapidly separate from the dragonfly airfoil surface, the ability to increase momentum and flow around the airfoil is lost, indicating the reason for significant fluctuations in the diagram of the aerodynamic coefficients of the dragonfly airfoil.

According to  Fig. 20, this incredible two-dimensional complexity evolves in the boundary layer. Thus, this is the boundary layer that flows over the flat or smooth part of NACA4412 and dragonfly airfoils. The developing and thickening of the boundary layer at the trailing edge and faster upper flow with slower lower flow create a Kármán vortex street, as shown in  Fig. 19.

According to Kolmogorov’s turbulence theory^42^, energy-containing and dissipation ranges, the turbulence kinetic energy (TKE) at the bottom surface of NACA4412 at the beginning of the downstroke are more intense than that of the dragonfly airfoil at $Re=5\times 10^4$. This energy forms a stronger vortex in NACA4412; this vortex is transferred to the airfoil’s trailing edge. It gets dissolved and enlarged by mixing with the high-speed flow of the upper surface.

From time *t*/*T*=1/4 onwards, midway downstroke, signs of increasing TKE are observed in the dragonfly airfoil on the leading edge. While at the leading edge of NACA4412, this increase in energy is not observed at all. In the dragonfly airfoil, this increase in TKE on the leading edge continues until *t*/*T*=3/4, and the vortex produced at the leading edge helps increase the vertical force and decrease the horizontal force in the dragonfly airfoil. By transferring this energy downstream and separating it from the trailing edge, the vertical force drops, and the drag force rises. At *t*/*T*=5/8 time, the TKE on the bottom of the leading edge in NACA4412 increases and continues up until the end of the upstroke, assisting in the formation of a high-energy counterclockwise vortex.

## Conclusion

In this study, the influence of the ground effect on the aerodynamic efficiency of a 2D flapping dragonfly airfoil was investigated and compared to the NACA4412 airfoil. An incompressible 2D finite volume CFD solver was employed for URANS. The turbulence model was implemented using the Transition SST model. The clearance effects between airfoil and wall, *St* and *Re*, were investigated systematically. The results indicated a direct relationship between the $C_l$/$C_d$ of the airfoil and the ground effect. At $Re=5\times 10^4$, by increasing St from 0.2 to 0.6, the values of $C_l$/$C_d$ decreased from 10.34 to 2.1 and 3.22 to 1.8 for NACA4412 and dragonfly airfoils, respectively. As a result, the $C_l$/$C_d$ of the NACA4412 airfoil was better than that of the dragonfly airfoil, especially at low oscillation frequency. The efficiency difference between the two airfoils at *St*=0.6 was approximately 94%, indicating that the $C_l$/$C_d$ difference decreased substantially with increasing frequency. The results of $Re=5\times 10^3$ showed that dragonfly airfoil was better $C_l$/$C_d$ in all frequencies compared to NACA4412 airfoil. The bigger and stronger LEV on NACA4412 airfoil in St = 0.2 with $Re=5\times 10^4$ was due to the highest $C_l$/$C_d$. This result can be expressed as follows: high Re and low *f*, a cambered airfoil would have enough efficiency to avoid wasting energy. Dragonfly airfoil is not able to absorb energy to produce more aerodynamic forces due to producing lower pressure circulators than the NACA4412 airfoil. Finally, the NACA4412 airfoil has a better lift coefficient than dragonfly airfoil at $Re=5\times 10^3$ and $5\times 10^4$; however, the aerodynamic performance of dragonfly airfoil is much better at $Re=5\times 10^3$ due to its low drag coefficient.

## Data Availability

The datasets used and/or analyzed during the current study are available from the corresponding author on reasonable request.

## Roman symbols

*c*: Chord length [*m*]
*f*: Flapping motion frequency [1/*s*]
$$k=\frac{\omega c}{ U_{\infty }}$$: Reduced frequency
$$C_l$$: Lift coefficient
$_{\overline{C_l}}$: Mean lift coefficient
$$C_d$$: Drag coefficient
$_{\overline{C_d}}$: Mean drag coefficient
$$\frac{C_l}{C_d}$$: Aerodynamic performance
$h_0$: Initial distance from the ground
$$h_1$$: Plunging amplitude [*m*]
*P*: Pressure [*Pa*]
*Re*: Reynolds number
$Re_{\theta t}$: Transition onset Reynolds number
$$St=\frac{kh_1}{\pi c}$$: Strouhal number
*t*: Flow time [*s*]
*T*: Flapping period [*s*]
$$U_{\infty }$$: Freestream velocity [*m*/*s*]

## Greek symbol

$\alpha$: Angle of attack [*deg*]
$\alpha _0$: Incidence angle of airfoil [*deg*]
$\alpha _1$: Pitching amplitude [*deg*]
$$\gamma $$: Intermittency factor
$\omega$: Angular velocity,2*f*, [rad/s]
$\phi$: Phase angle [*deg*]
$\rho$: Density of air [$kg/m^3$]
$\varepsilon$: Turbulence dissipation rate [$m^2/s^3$]

## Abbreviation

*AoA*: Angle of attack
*CW*: Clockwise
*CCW*: Counterclockwise
*FVM*: Finite volume method
$IB-LBM$: Immersed boundary-lattice Boltzmann method
*LE*: Leading edge
*LEV*: Leading edge vortex
*MAV*: Micro air vehicles
*OGE*: Out of ground effect
*TE*: Trailing edge
*TEV*: Trailing edge vortex
*TKE*: Turbulence kinetic energy
*URANS*: Unsteady Reynolds averaged Navier-Stokes
